# An analysis of pharmacy workforce capacity in Saudi Arabia

**DOI:** 10.3389/fphar.2023.1219528

**Published:** 2023-08-03

**Authors:** Dalia Almaghaslah

**Affiliations:** Department of Clinical Pharmacy, College of Pharmacy, King Khalid University, Abha, Saudi Arabia

**Keywords:** workforce intelligence, pharmacy, Saudi Arabia, workforce capacity, supply and demand

## Abstract

**Background:** Previous reports have highlighted the core issues with the intelligence of the national pharmacy workforce reliance on non-native pharmacists, despite the increasing supply of local pharmacy graduates; limited participation of female pharmacists in the workforce; and inadequate proportions of pharmacists in primary healthcare centres, resulting in pharmaceutical services being provided without a pharmacist’s supervision.

**Methods:** The current study used a retrospective cross-sectional design and data was collected from January to May 2023. Data was retrieved from the Health Statistics Yearbook, 2017, 2018, 2019, 2020 and 2021. The data included the total number of pharmacists, the number of Saudi pharmacists, the number of foreign pharmacists, the distribution of the proportions of male and female pharmacists, and the distribution of the workforce by region, sector and nationality. Data was also obtained relating to pharmacy education, including the number of pharmacy colleges, the number of pharmacy students and the number of pharmacy graduates.

**Results and conclusion:** The capacity of the pharmacy workforce, meaning the number of pharmacists per 10,000 population, fluctuated between 2017 and 2021, with the lowest number being in 2020, a mere 7.9 pharmacists per 10,000 population. However, in 2021, the overall density of pharmacists increased to (9.04), which is above the global average (7.36 per 10,000 population). The proportion of women working in the pharmacy profession increased from 12% to 22%, and in community pharmacies from 0.3% to 7.2%, between 2016 and 2021. Another issue that still exists is a lack of proportionate and imbalance in the distribution of the pharmacy workforce across the regions. The renationalisation initiatives increased the overall proportion of Saudi pharmacists to 39% in 2021, compared to 22% in 2016. There is a need for a policy mechanism that will overcome the identified issues.

## 1 Introduction

Achieving universal Health Coverage and Sustainable Development Goals requires investment in the health workforce (Meilianti et al., 2022; Al-Haqan et al., 2020; Almaghaslah and Alasayari, 2022). However, health systems around the globe have been facing difficulties with workforce capacity and accessibility (Bates et al., 2023). The pharmacy workforce, the third largest healthcare cadre and the most accessible healthcare profession, is still a part of these challenges (Almaghaslah, 2022). Strategies to overcome these difficulties differ among countries. The pharmacy profession in Saudi Arabia has been reformed to deal with the identified challenges (Almaghaslah, 2022). Previous reports have highlighted the core issues with the intelligence of the national pharmacy workforce (Almaghaslah et al., 2019; Almaghaslah D. and Alsayari A., 2021; AlRuthia et al., 2018): reliance on non-indigenous pharmacists, despite the increasing supply of local pharmacy graduates (Almaghaslah et al., 2019); limited participation of female pharmacists in the workforce; the career pathway preferences of new Saudi pharmacy graduates, favouring hospital pharmacies in spite of the limited job vacancies within this sector; and community pharmacies being the most in-demand pharmacy sector (Almaghaslah et al., 2021); inadequate proportions of pharmacists in primary healthcare centres, resulting in pharmaceutical services being provided without pharmacists’ supervision (Almaghaslah et al., 2019).

The government in Saudi Arabia has issued several reforming initiatives to cope with the important challenges (Almaghaslah Dalia and Alsayari Abdulrhman, 2021). Healthcare workforce localisation, including at pharmacies, was reinforced in the 2030 healthcare vision (Almaghaslah D. and Alsayari A., 2021; Almaghaslah et al., 2022a; Albejaidi and Nair, 2019). The focus was on the largest pharmacy employment sectors, community pharmacies and the pharmaceutical industry. Gradual Saudistion of the profession was imposed at 20% by 2020 and 30% by 2021 (Almaghaslah, 2022; Almaghaslah Dalia and Alsayari Abdulrhman, 2021).

The participation of women in the pharmacy job market was encouraged and facilitated by easing the restrictions that had previously prevented them from working in the community setting (Almaghaslah et al., 2021; Alhazmi et al., 2022). This helped create more employment opportunities for females and improved gender equity as well as localisation of the workforce (Almaghaslah et al., 2022a). The government-private partnership has been strengthened by increasing the participation of the private sector in employing national healthcare cadres and the delivery of healthcare services. Community pharmacies have been a major provider of pharmaceutical services to the government hospitals and primary healthcare centres (Almaghaslah et al., 2022b). An electronic prescribing service, Wasanty, was established to link the Ministry of Health centres to community pharmacies and hence gradually replace local pharmacies within the government premises, thus ensuring that pharmaceutical services are delivered by authorised and competent pharmacists (Almaghaslah et al., 2022b).

More recent legislation was directed at education institutions for the healthcare disciplines (Ministry of Education, 2022). It required doubling the intake of healthcare students for 5 years. It also required monitoring employment of their graduates (Ministry of Education, 2022). This initiative aimed to meet the demand for these specialities in the job market, further nationalising the healthcare professions, and improving the output competency and performance of the university education system (Ministry of Education, 2022).

Considering the ongoing efforts and the reforming legislation affecting pharmacy education, employment laws, accessibility and quality of pharmaceutical services. Monitoring of the pharmacy workforce is needed to assess the effects of these changes in pharmacists’ capacity, workforce gender distribution and workforce planning. This study aims to provide insight and obtain evidence to inform pharmacy workforce planning and policy development in Saudi Arabia.

## 2 Methods

Various modelling system approaches are available. The complexity of these approaches varies. The simplest deterministic model is based on a predictable relationship between supply and demand. The four main categories are: needs-based, utilisation, human resources to population ratio and target settings (Almaghaslah et al., 2019).

Previous studies have identified the main issues with the intelligence of a pharmacy workforce, including workforce distribution, the economic status of the country, capacity building and gender distribution.

This paper presents a follow-up on a previously published work using the same approach, i.e., a simple integrated model, workforce supply/output as density. This basic approach is adopted from the FIP global pharmacy workforce reports. Density is presented as the number of pharmacists per 10,000 population. It takes into consideration population growth as a measure for demand. Due to the limited available data, a simple supply rather than supply and demand model was chosen. Data was retrieved from the Health Statistics Yearbook, 2017, 2018, 2019, 2020 and 2021 (Ministry of Health, 2021). Data included the total number of pharmacists, the number of Saudi pharmacists, the number of foreign pharmacists, the distribution of male and female pharmacists, and the distribution of the workforce by region, sector and nationality. Data was also obtained relating to pharmacy education, including the number of pharmacy colleges, the number of pharmacy students and the number of pharmacy graduates (Ministry of Education, 2023). The community pharmacy sector, the largest sector, was analysed in terms of gender balance and nationality proportions. Pharmacists employed in primary healthcare centres were analysed to assess the effects of shifting pharmaceutical services to local community pharmacies, looking at the supply side function trends, including the number of pharmacy colleges and the number of students.

The current study used a retrospective cross-sectional design, and the data was collected from January to May 2023. Pharmacy education data from 2017 to 2023 was retrieved and other data between 2017 and 2021 was retrieved. The retrieved data was cleared, entered and analysed using Microsoft Excel. The results are described in terms of frequencies and percentages. Ethical approval was not needed, as data was available in the public domain.

## 3 Results

### 3.1 Pharmacy workforce supply

#### 3.1.1 Pharmacy education

**FIGURE 1:**
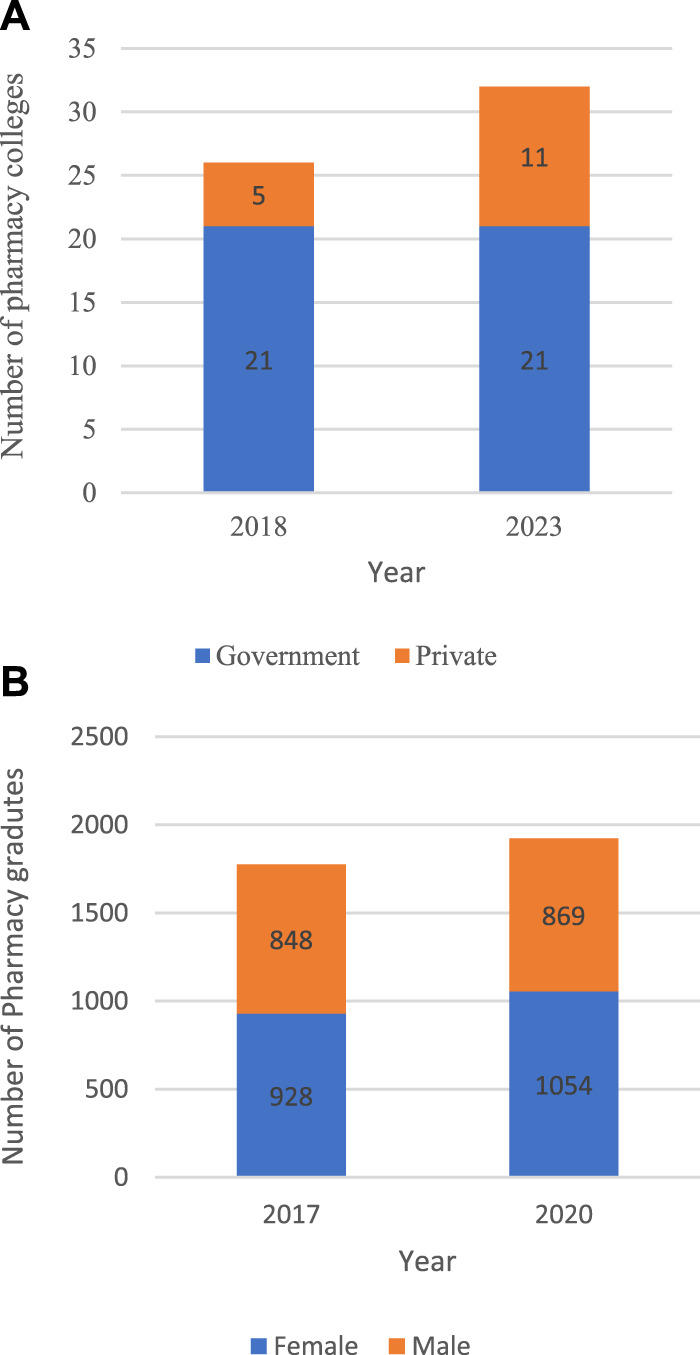
Number of pharmacy colleges by sector **(B)**. Number of pharmacy graduates by gender.

The number of government pharmacy colleges remained constant from 2016 to 2023 at 21 colleges, but the number of private schools increased from 5 in 2016 to 11 in 2023. The total number reached 31 pharmacy colleges in 2023, as shown in Figure 1A. As a result of increasing the number of pharmacy schools, the number of graduates increased to 1,923 in 2020, compared to 1,776 in 2017, with the proportion of females remining higher than their male counterparts, as illustrated in Figure 1B.

#### 3.1.2 Pharmacy workforce capacity

**FIGURE 2:**
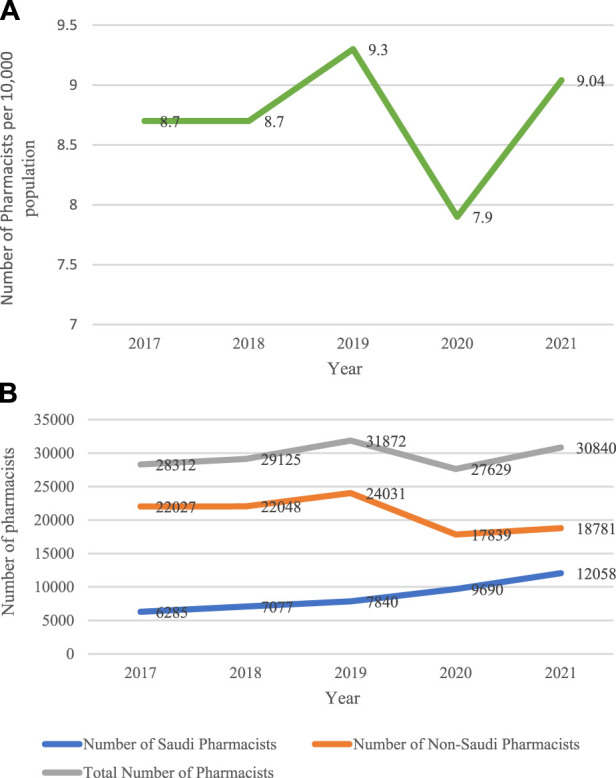
Number of pharmacists per 10,000 population **(B)**. Number of pharmacists by nationality from 2017 to 2021.

The total number of pharmacists remained constant at 8.7 from 2017 to 2,108. It peaked at 9.3 in 2019, then it dropped to the lowest (7.8) in 2020. It rose again to 9.04 pharmacists in 2021, as shown in Figure 2A. Workforce nationality analysis indicated that the number of Saudi pharmacists continued to rise steadily from 6,285 in 2017 to 7,077 in 2,108, and it reached 7,840 pharmacists in 2019. The number continued to increase from 9,690 in 2020 to reach its maximum of 12,058 pharmacists in 2021. On the other hand, the number of non-indigenous pharmacists fluctuated between 2017 and 2019 with 22,027, 22,048, and 24,031, respectively. It declined to the minimum in 2020 with 17,838, then rose slightly to reach 18,781, as illustrated in Figure 2B.

## 4 Pharmacy workforce distribution by region, gender and sector

### 4.1 Workforce distribution by region

**FIGURE 3:**
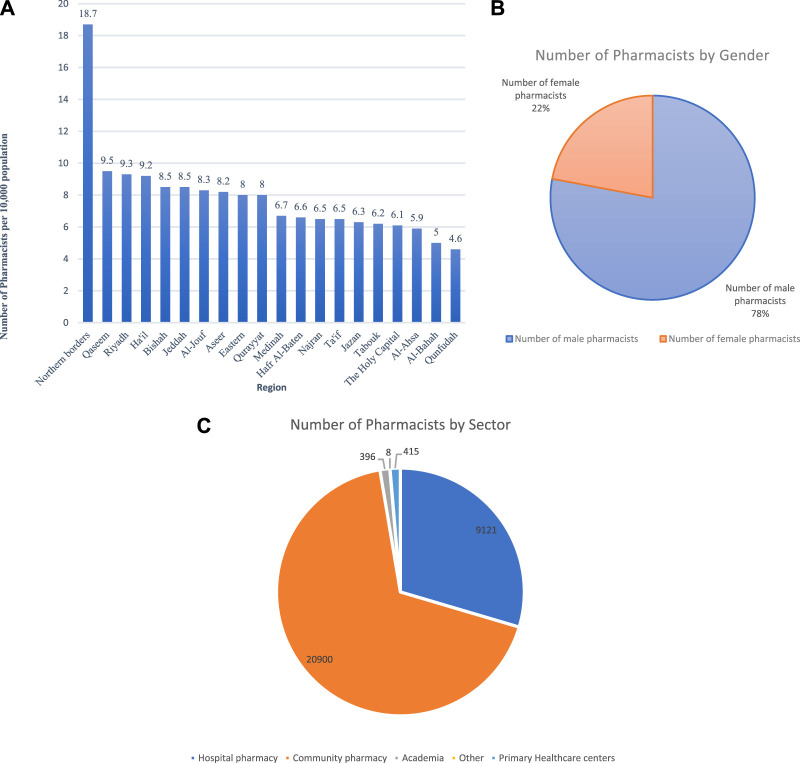
**(A)** Pharmacy workforce distribution by region per 10,000 **(B)**. Workforce distribution by gender **(C)**. Workforce distribution by sector.

Analysis of pharmacy workforce distribution by region shows that there is inequitable distribution of pharmaceutical manpower across the country. Some regions were found to have the highest proportions of pharmacists per 10,000 inhabitants, such as Northern Borders and Qaseem with 18.7 and 9.5 pharmacists, respectively. On the other hand, some regions—such as Albaha and Qunfudah—were reported to have the lowest proportions, with 5 and 4.6 pharmacists per 10,000 population, respectively as shown in Figure 3A.

### 4.2 Workforce distribution by gender

Figure 3B shows pharmacy workforce distribution by gender. The majority of the pharmacists (24,042, 78%) were males and (6,798, 22%) were females.

### 4.3 Workforce distribution by sector

Community pharmacies form the largest employment sector, with 20,900 pharmacists (67.8%). Hospital pharmacies form the second largest sector with 9,121 pharmacists making up (29.6%) of the total workforce. Primary healthcare centres employ (1.3%) 415 pharmacists. Academia makes up only (1.3%), with 396 academics, while others (8, 0.03%) are employed in various authorities, such as airlines and ministries, as shown in Figure 3C.

*Data on pharmacists working in the pharmaceutical industry is not available.

## 5 Pharmacy workforce in community settings and primary healthcare centres

**FIGURE 4:**
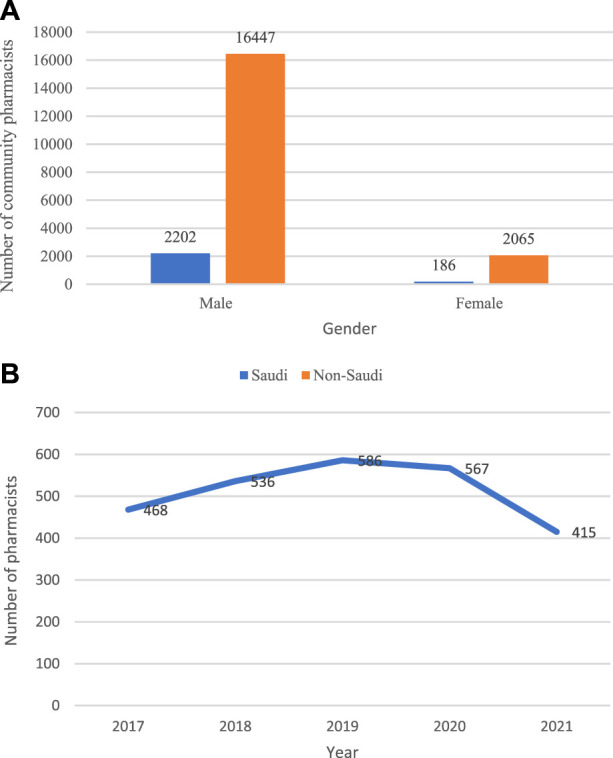
**(A)** Community pharmacist distribution by gender and nationality **(B)**. The number of pharmacists working in primary healthcare centres.

The total number of pharmacists working in community settings was 20,900. The vast majority of community pharmacists are males (18,649, 89.2%), of which (16,447, 78.7%) are non-Saudi. The number of women working in community settings (2,251) makes up only 10.8% of the total. However, the number of Saudi female pharmacists (2,065, 9.9%) is much higher than the number of non-Saudis (186, 0.9%), Figure 4A.

The number of pharmacists working in primary healthcare centres increased from 468 to 536 between 2017 and 2018. It peaked in 2019 with 586, then started to decline, reaching 567 in 2020 and decreasing further to reach its minimum of 415 in 2021, Figure 4B.

## 6 Pharmacy workforce renationalisation by sector and gender

**FIGURE 5:**
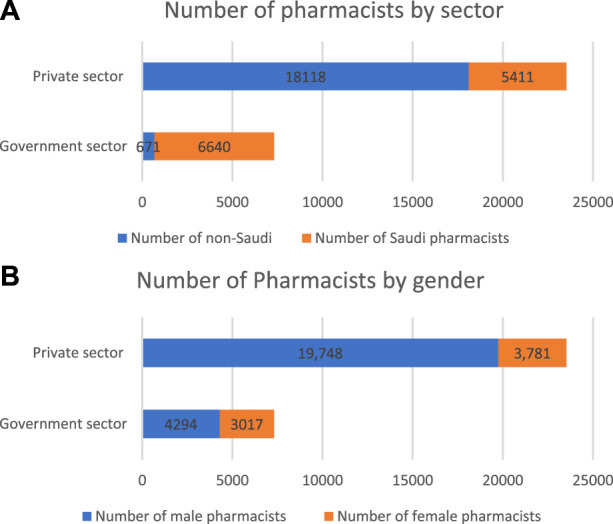
Pharmacist workforce distribution in the private and government sectors by **(A)** nationality and **(B)** gender.

Figure 5A illustrates the pharmaceutical workforce distribution by nationality in the government and the private sectors. The workforce nationality data indicates higher ratios of nonnational pharmacy personnel. Of the total pharmacy workforce of 30,840, 18,789 (60.9%) are nonindigenous. The government sector has achieved a higher renationalisation ratio with 6,640 (90.8%) compared to 3,781 (23%) in the private sector.

Figure 5B shows the gender distribution of pharmaceutical human resources. Females make up 22% (*n* = 6,798) of the total workforce (*n* = 30,840). The percentage of females working in the private sector (3,781, 55.6%) is slightly higher than those working for the government (3,017, 44.4%). The private sector includes hospitals, community pharmacies and other institutions.

## 7 Discussion

The current study aimed to provide an update on the status of the pharmacy workforce in Saudi Arabia. Effective and regular monitoring of the human resources is essential for the successful planning of the workforce. In this study, several supply parameters were evaluated.

The data indicated that the number of private pharmacy schools continues to rise, despite the previous recommendations by the SCFHS to stop establishing new pharmacy schools, both private and government, to prevent oversupply (Almaghaslah et al., 2019). The number of government colleges remined the same, but the intake of students continued to rise as the national renationalising plan for the health workforce, including pharmacies, urged pharmacy colleges to double the intake of pharmacy students. As a result, the supply side of local pharmacists is gradually replacing international pharmacists, as the percentage of Saudi pharmacists reached 39% in 2021 compared to 22% in 2017.

The capacity of the pharmacy workforce—i.e., the number of pharmacists per 10,000 population—fluctuated between 2017 and 2021, with the lowest point being in 2020 with 7.9 pharmacists per 10,000 population. A workforce by nationality comparison showed that, during 2020 and the COVID-19 pandemic, a sharp decrease was the result of the decline in the numbers of non-indigenous pharmacists, which affected the overall number of pharmacists. This finding raises explicit concerns about workforce sustainability in times of crisis. During COVID, the country’s global travel restrictions to contain the spread of the virus affected the availability of non-national pharmacists (Almaghaslah and Alsayari, 2020; Almaghaslah et al., 2020). However, in 2021 the overall density of pharmacists increased to (9.04), which is above the global average (7.36 per 10,000 population), indicating that there is an equilibrium between supply and demand (Meilianti et al., 2022).

Another issue that still exists is the disproportion and imbalance in the distribution of the pharmacy workforce across the regions. More urbanised areas tend to have a higher density of pharmacists than rural and remote areas. This issue has been identified worldwide, especially in lower- and middle-income countries such as Nigeria, Indonesia, Tanzania and Ghana (Meilianti et al., 2022; Ekpenyong et al., 2018). Strategies that would support the recruitment and retention of pharmacists in the underserved regions—particularly Al-Ahsa, Al-Bahah, and Qunfudah—should be introduced.

The number of pharmacists working in primary healthcare centres has further decreased as a result of shifting services delivery to the community pharmacy sector, through the e-prescribing system Wasanty that facilitated the private-government partnership in services delivery (Almaghaslah et al., 2022b). Hence, the quality of services should have improved, as it was previously found that pharmaceutical services in primary healthcare centres were run without pharmacist’s supervision (Almaghaslah et al., 2019). This has also created more job opportunities for local pharmacy graduates in community settings.

The distribution of pharmacists across sectors shows that community pharmacies remain the largest employment sector for pharmacists in the country, with 20,900 (67.8%) employed in the field and 9,121 (30%) employed in institutional settings. Globally, community pharmacies employ around 55% and hospitals employ18% of the total workforce (Bates et al., 2016).

In contrast to the global trend of greater proportions of female pharmacy workforce, where women make up more than 65% of the total workforce in some regions, the female pharmacy workforce in Saudi Arabia made up only 22% of the total in 2021 (Bates et al., 2016).

Easing the restrictions that previously prevented female pharmacists from working in community settings has created job opportunities for women in order to fulfil the 2030 Vision that aimed to increase the participation of females in the job market (Almaghaslah et al., 2021; Al-Hanawi et al., 2019). Overall, the proportion of women working in the pharmacy profession increased from 12% to 22%, and in community pharmacies from 0.3% to 7.2%, between 2016 and 2021.

The renationalisation initiatives have increased the overall proportion of Saudi pharmacists to 39% in 2021 compared to 22% in 2016. Analysis of pharmacists’ distribution in the public and private sectors indicated that the public sector achieved 90.8%, whereas the private achieved 23% localisation ratios, respectively. As a result, the proportion of non-indigenous pharmacists has gradually decreased by replacing them with national pharmacists following the 2030 Vison that aims at nationalising the profession (Albejaidi and Nair, 2021). Hence, creating more job opportunities for Saudi pharmacist has been incrementally achieved. Another positive impact is that the sustainability of the workforce in likely to be higher, as the number of Saudi pharmacists did not change during the COVID-19 pandemic. However, with an increasing number of pharmacy schools and pharmacy graduates, the private sector would be required to impose strategic solutions to achieve the Saudistion ratio to accommodate the increasing supply of the national pharmacy workforce.

### 7.1 Study limitations

Limited pharmacy workforce data is available globally and Saudi Arabia is no different. Data in this study was extracted from two sources: Health Statistics Yearbook 2017, 2018, 2019, 2020 and 2021 as well as the Ministry of Education website. These data source do not consider the demand factors, such as mortality rate, life expectancy, disease pattern, as well as migration. They do not provide information about pharmacy workforce age, pharmacists working in non-health related sectors, including the pharmaceutical industry, academia and marketing, the inactive pharmacy workforce, or exit from the workforce due to retirement, death and other reasons. These data source use the pharmacists-to population ratio as a measure for workforce evaluation, which does not provide an in-depth evaluation of workforce productivity and working patterns. Another possible limitation is that the most recent data available was from the Health Statical Yearbook 2021 and hence there is a 2-year gap.

## 8 Conclusion

The study findings indicate that the capacity of the pharmacy workforce is comparable to the global average. However, widespread variance in the distribution of the workforce across the regions still exists. The participation of female pharmacists in the job market has increased over the past 5 years, but is still far below the rest of the world. The proportions of the Saudi pharmacy workforce have almost doubled over the last 5 years, but there is a need for a policy mechanism that will promote localisation of the workforce, especially with the ongoing increased output of local pharmacy graduates.

## Data Availability

The data presented in the study are available: https://figshare.com/s/045cd7f00f013d3d7c63.
